# Supplementary material for: Minute-scale oscillatory sequences in medial entorhinal cortex
Source: Nature. 2023 Dec 20;625(7994):338–44. doi: 10.1038/s41586-023-06864-1 (PMC10781645; doi:10.1038/s41586-023-06864-1)
Supplement: Supplementary file 1 — Reporting Summary [file 41586_2023_6864_MOESM1_ESM.pdf]

nature portfolio

Corresponding author(s): Soledad Gonzalo Cogno
May-Britt Moser
Edvard Moser

Last updated by author(s): Sep 26, 2023

Reporting Summary

Nature Portfolio wishes to improve the reproducibility of the work that we publish. This form provides structure for consistency and transparency in reporting. For further information on Nature Portfolio policies, see our Editorial Policies and the Editorial Policy Checklist.

Statistics

For all statistical analyses, confirm that the following items are present in the figure legend, table legend, main text, or Methods section.

n/a	Confirmed	
☐	☒	The exact sample size (*n*) for each experimental group/condition, given as a discrete number and unit of measurement
☐	☒	A statement on whether measurements were taken from distinct samples or whether the same sample was measured repeatedly
☐	☒	The statistical test(s) used AND whether they are one- or two-sided<br>*Only common tests should be described solely by name; describe more complex techniques in the Methods section.*
☐	☒	A description of all covariates tested
☐	☒	A description of any assumptions or corrections, such as tests of normality and adjustment for multiple comparisons
☐	☒	A full description of the statistical parameters including central tendency (e.g. means) or other basic estimates (e.g. regression coefficient) AND variation (e.g. standard deviation) or associated estimates of uncertainty (e.g. confidence intervals)
☐	☒	For null hypothesis testing, the test statistic (e.g. *F*, *t*, *r*) with confidence intervals, effect sizes, degrees of freedom and *P* value noted<br>*Give P values as exact values whenever suitable.*
☒	☐	For Bayesian analysis, information on the choice of priors and Markov chain Monte Carlo settings
☒	☐	For hierarchical and complex designs, identification of the appropriate level for tests and full reporting of outcomes
☐	☒	Estimates of effect sizes (e.g. Cohen's *d*, Pearson's *r*), indicating how they were calculated

Our web collection on statistics for biologists contains articles on many of the points above.

Software and code

Policy information about availability of computer code

Data collection

MESc, versions 3.3 and 3.5, Femtonics, Hungary (2p Imaging); ZEN, Version 3 (blue edition), Carl ZEISS, Germany (confocal microscopy), SpikeGLX (https://billkarsh.github.io/SpikeGLX) version 20201103, Imec phase30 version 3.31, Motive (OptiTrack) version 2.2.0, MATLAB (MathWorks) version r2020a

Data analysis

Commercial software: MATLAB (MathWorks) versions r2019b, r2020a and r2021b, Python version 3.7, Imaris versions 9.8.0 and 9.8.2, Bitplane (processing of histological images).

Open-source Python codes:
- Suite2P: https://github.com/MouseLand/suite2p
- Kilosort (version 2.5): https://github.com/MouseLand/Kilosort

Open-source Matlab codes:
- UMAP version 1.3.4: https: //se.mathworks.com/matlabcentral/fileexchange/71902-uniform-manifold-approximation-and-projection-umap
- Toolbox for Dimensionality Reduction, available in: https://lvdmaaten.github.io/drtoolbox/
- Circular Statistics Toolbox version 1.21.0.0: https://se.mathworks.com/matlabcentral/fileexchange/10676-circular-statistics-toolbox-directional-statistics
Paper: https://www.jstatsoft.org/article/view/v031i10

For manuscripts utilizing custom algorithms or software that are central to the research but not yet described in published literature, software must be made available to editors and reviewers. We strongly encourage code deposition in a community repository (e.g. GitHub). See the Nature Portfolio guidelines for submitting code & software for further information.

Data

Policy information about availability of data

All manuscripts must include a data availability statement. This statement should provide the following information, where applicable:

- Accession codes, unique identifiers, or web links for publicly available datasets
- A description of any restrictions on data availability
- For clinical datasets or third party data, please ensure that the statement adheres to our policy

The datasets generated during the current study will be available after publication, on EBRAINS.

Field-specific reporting

Please select the one below that is the best fit for your research. If you are not sure, read the appropriate sections before making your selection.

☒ Life sciences ☐ Behavioural & social sciences ☐ Ecological, evolutionary & environmental sciences

For a reference copy of the document with all sections, see nature.com/documents/nr-reporting-summary-flat.pdf

Life sciences study design

All studies must disclose on these points even when the disclosure is negative.

Sample size	Samples included all available cells.
Data exclusions	Cells with very low signal-to-noise ratio (below 4) were excluded because of their unsuitability for the performed analyses.
Replication	For all animals included in the study, in the results text we indicate for each animal either the experimental sessions or the fraction of experimental sessions in which the effect was found. From 5 MEC calcium imaging animals, ultraslow oscillatory sequences were observed in 4 animals. From 2 MEC Neuropixels animals, ultraslow oscillatory sequences were observed in the 2 animals.
Randomization	The study did not involve any experimental subject groups; therefore, random allocation did not apply and was not performed.
Blinding	The study did not involve any experimental subject groups; therefore, experimenter blinding did not apply and was not performed.

Reporting for specific materials, systems and methods

We require information from authors about some types of materials, experimental systems and methods used in many studies. Here, indicate whether each material, system or method listed is relevant to your study. If you are not sure if a list item applies to your research, read the appropriate section before selecting a response.

Materials & experimental systems

n/a	Involved in the study	
☒	☐	Antibodies
☒	☐	Eukaryotic cell lines
☒	☐	Palaeontology and archaeology
☐	☒	Animals and other organisms
☒	☐	Human research participants
☒	☐	Clinical data
☒	☐	Dual use research of concern

Methods

n/a	Involved in the study	
☒	☐	ChIP-seq
☒	☐	Flow cytometry
☒	☐	MRI-based neuroimaging

Animals and other organisms

Policy information about studies involving animals; ARRIVE guidelines recommended for reporting animal research

Laboratory animals	C57/Bl6 mice, male. Age: 3-5 months for one group (22-36g during the recordings), 0-2 months for another group (15-20g during the recordings).
Wild animals	None
Field-collected samples	None
Ethics oversight	Protocols approves by Norwegian Animal Welfare Act and the European Convention for the Protection of Vertebrate Animals used

Ethics oversight | for Experimental and Other Scientific Purposes, Permit numbers 18011 and 29893.

Note that full information on the approval of the study protocol must also be provided in the manuscript.
